# Hydrogen sulfide suppresses ghrelin secretion in vitro and delays postprandial ghrelin secretion while reducing appetite in mice

**DOI:** 10.14814/phy2.13870

**Published:** 2018-10-07

**Authors:** Erik Slade, Laura Williams, Jeffrey Gagnon

**Affiliations:** ^1^ Department of Biology Laurentian University 935 Ramsey Lake Road Sudbury Ontario Canada P3E2C6

**Keywords:** Cell biology, gastrointestinal tract, ghrelin, hormone secretion

## Abstract

Ghrelin is a stomach‐derived hormone that regulates several metabolic functions including growth hormone release, appetite, adiposity, and gastric motility. Nutrients, the autonomic nervous system, and other metabolic hormones have all been implicated in the regulation of ghrelin secretion. Despite this, ongoing efforts to develop modulators of ghrelin secretion in human diseases are still underway. Hydrogen sulfide (H_2_S) is a gaseous signaling molecule that is produced both endogenously in many tissues and by the gut microbiome. H_2_S has established roles in cardiovascular and immune health, however, more recently H_2_S has been implicated in the regulation of metabolic hormone secretion. We hypothesized that H_2_S is able to directly regulate ghrelin secretion and in turn, regulate appetite. We first demonstrated that GYY4137 (an H_2_S donor molecule) directly suppresses ghrelin secretion in rat primary gastric culture, in part through the activation of the protein kinase B (AKT) pathway. We then demonstrated the colocalization of ghrelin‐positive gastric cells with the H_2_S producing enzyme cystathionine‐*γ*‐lyase (CSE). While GYY4137 suppressed ghrelin secretion, inhibition of CSE caused a stimulation in ghrelin secretion in primary gastric culture. In mice, GYY4137 treatment prolonged the postprandial drop of circulating ghrelin and caused reduced food consumption up to 4 h after treatment. These results demonstrate for the first time a role for H_2_S in the regulation of ghrelin and appetite. Modulating H_2_S levels may be a novel approach to regulate ghrelin secretion in the treatment of metabolic diseases.

## Introduction

Ghrelin is a stomach‐derived hormone that has metabolic functions throughout the body primarily in energy metabolism (Kojima et al. 1999). Ghrelin has been shown to stimulate appetite, stimulate growth hormone secretion, promote adiposity, and stimulate gut motility (Müller et al. 2015). As ghrelin plays many important roles in metabolism, understanding how this hormone is regulated is of key importance in metabolic health and disease. It is well documented that plasma ghrelin levels are highest prior to food consumption and are suppressed *postprandially*. Accordingly, the circadian rhythm of circulating ghrelin fluctuates around meal times, suggesting that ghrelin plays an important role in meal cues and postmeal metabolism. There are several pathways involved in regulating ghrelin secretion. The type of macronutrient consumed will yield different levels and duration of ghrelin suppression with proteins and fats manifesting the longest suppression (Foster‐Schubert et al. 2008; McGavigan et al. 2015). The autonomic nervous system has also been implicated in regulating ghrelin secretion (Hosoda and Kangawa 2008; Gagnon and Anini 2012). In addition, our research and others has implicated metabolic hormones, including insulin and GLP‐1 in the regulation of ghrelin (Hagemann et al. 2007; Gagnon and Anini 2012).

A novel pathway in the regulation of ghrelin may be through gas signaling molecules including hydrogen sulfide (H_2_S). H_2_S is an endogenously produced (through several enzymatic pathways) gaseous signaling molecule. Research on H_2_S has largely focused on its cardiovascular effects through vascular tone (Wang 2012), with most studies indicating that H_2_S is a vasodilator with cardioprotective, anti‐apoptotic, and anti‐inflammatory effects, reviewed in (Salloum 2015). Interestingly, several groups have demonstrated a role for H_2_S in the regulation of insulin secretion, reviewed in (Pichette and Gagnon 2016). In endocrine beta cells, H_2_S has been shown to modulate a variety of ion channels and cellular kinases (Pichette and Gagnon 2016). Recently, our group demonstrated a role for H_2_S in the stimulation of the gastric hormone glucagon like peptide 1 (GLP‐1). However, the role H_2_S plays on other metabolic hormones, including ghrelin, remains to be determined. When examining the production of H_2_S from tissues in mice, the stomach was found to both express the H_2_S‐producing enzyme cystathionine‐*γ*‐lyase (CSE) and produce detectable levels of this gas (Fiorucci et al. 2005). Since H_2_S is produced in the stomach (the site of 70–80% of ghrelin production (Jeon et al. 2004)), and is already known to regulate other gastric hormones (Yang et al. 2005; Pichette et al. 2017), we hypothesized that H_2_S would play a role in the regulation of gastric ghrelin. We investigated this hypothesis through a combination of cell and animal‐based methods. In vitro, using primary rat stomach culture, we tested the effect of H_2_S donors and CSE inhibitors on ghrelin secretion and cell signaling. In vivo, we examined the effect of these compounds on postprandial ghrelin suppression and appetite in mice.

## Material and Methods

### Animals

Experiments were performed following the guidelines outlined by the Canadian Council on Animal Care guide to the Care and Use of Experimental Animals (CCAC, Ottawa, ON: Vol. 1, 2nd edition, 1993: Vol. 2, 1984) (Olfert 2017). Animal Protocols were approved by the Laurentian University Animal Care Committee. Pregnant female Sprague–Dawley rats as well as male and female wild‐type C57BL/6 mice aged 7–8 weeks were purchased from Charles River Laboratories (St. Constant, Quebec). The dams and pups were housed together until pups were sacrificed on postnatal day 8 for primary culture preparation. The mice were all singly housed in standard cages. All rodents were maintained on a 12‐h light/dark cycle in the Paul Field Animal Care Facility at Laurentian University.

### Primary culture preparation

All cell culture media and reagents, unless otherwise stated, were obtained from Sigma Aldrich (Oakville, ON, Canada). Primary cell culture was prepared from male and female rat stomachs postnatal day 8 as indicated in (Gagnon and Anini 2012). Briefly, stomachs from each litter were separated into three equal portions, extracted, rinsed and enzymatically digested twice with collagenase. The final pellet was resuspended in 10 mL of culture media (low glucose DMEM, 10% fetal bovine serum (FBS), 1% Streptomycin/Penicillin (SP)). Cells were plated in 6 or 12 well culture plates for ghrelin secretion, western blotting, or immunocytochemistry as indicated below.

### Ghrelin secretion experiments

Secretion experiments were conducted using rat stomach primary culture seeded into 6‐well culture plates (2,000,000 cells per well). Twenty‐four hour after initial plating the media were removed and replaced using a culture media containing 50 *μ*mol/L octanoic acid. After an additional 24 h, cells were washed with wash buffer then incubated at 37°C for 4 h with treatments dissolved in 2 mL secretion media (low glucose DMEM 0.5% FBS 1% SP). The treatments (Cayman Chemicals, Ann Armor, MI): H_2_S donor GYY4137, which releases H_2_S via hydrolysis reactions in solution, CSE inhibitor DL‐Propargyl Glycine (PPG) or phosphoinositide 3 kinase (PI3K) inhibitor LY294002 were dissolved in DMSO. Media with vehicle (DMSO) served as a control for baseline secretion and forskolin (which increases intracellular cAMP) were used as a positive control for acylated ghrelin secretion. Following the 4 h incubation, the media were collected and spun down at 1000*g* for 5 min to remove floating cell debris. The supernatant was acidified using trifluoroacetic acid (TFA) to a final concentration of 0.1% and stored at −20°C to prevent protease activity and loss of acylated ghrelin. The cell lysates were collected off the plate using a cell scraper in 500 *μ*L of an acidic lysis buffer (1 mol/L HCl, 1% TFA and 50 mmol/L NaCl) and sonicated for 10 sec on ice (Hosoda 2004). The lysate was spun down at 13,000*g* at 4°C. The supernatant was separated from cell debris and stored at −20°C. The media and lysates were subjected to hydrophobic reverse phase resin chromatography (C‐18 SepPak cartridges, Waters) according to manufacturer's instructions and eluted in 5 mL of 80% isopropanol in water containing 0.1% TFA. Samples were then dried in a vacuum concentrator. These dried samples were stored at −20°C until analysis using a commercial acylated ghrelin enzyme immunoassay kit described below. Cell viability was determined under similar experimental conditions using the neutral red uptake assay described in (Repetto et al. 2008).

### Western blot

Primary stomach cells were seeded into 6‐well culture plates (2,000,000 cells per well) and received treatments after 48 h as indicated above. On the day of treatment, cells were washed with wash buffer and treated with secretion media alone or secretion media containing 100 *μ*mol/L GYY4137 for 15 min at 37°C, 5% CO_2_. Each well received 100 *μ*L of Cytobuster cell lysis buffer (EMD Biosciences, Gibbstown, NJ) supplemented with protease and phosphatase inhibitor cocktails (Complete Mini/Phospho‐Stop; Roche Applied Science, Guelph, Ontario, Canada) and cells were collected, sonicated on ice (10 sec, Power 4), and centrifuged (5 min, 13,000*g*, 4°C). Protein concentration was measured using the Bradford protein method and the expression levels of phosphorylated AKT and total AKT were analyzed by Western blotting. 20 *μ*g of protein was loaded per lane on 4–12% acrylamide sodium dodecyl sulfate‐polyacrylamide gels and run at 180 V. The protein was then transferred for 1.5 h at 200 V onto a polyvinylidene fluoride membrane, blocked in 5% w/v milk powder and 0.1% Tween 20 TRIS buffered saline (TBST) for 45‐min, then incubated in primary antibodies (see Table 1) in TBST containing 5% BSA overnight. After 3 × 5 min washes in TBST, the membrane was incubated with a horseradish peroxidase‐conjugated secondary antibody at a concentration of 1:2000 for 1 h at room temperature. Signal was developed by exposing the membrane to Luminata Forte Western HRP Substrate (Millipore Corporation, Millerica, MA) for 2 min. Chemiluminescence signal was recorded using a BioRad Chemidoc XRS documentation apparatus and analyzed using QuantityOne program. Densitometry was used to analyze band intensity to compare phosphorylated AKT and total AKT protein expression between treatments.

**Table 1:** Antibody details

Peptide/protein target	Antigen sequence (if known)	Name of antibody	Manufacturer	Species raised in; monoclonal or polyclonal	Dilution used	RRID
Cystathionine *γ*‐lyase		Anti‐Cystathionase	Abcam	Rabbit; polyclonal	1/100	AB_2722603
Ghrelin		Anti‐Ghrelin	Abcam	Mouse; monoclonal	1/100	AB_941760
Phospho‐AKT (Ser473)	Ser473	Phosphor‐AKT (Ser473)	Cell Signaling Technology	Rabbit; monoclonal	1/1000	AB_2315049
AKT (pan)		Akt (pan) (C67E7) Rabbit mAb antibody	Cell Signaling Technology	Rabbit; monoclonal	1/1000	AB_915783
Rabbit IgG		Anti‐Rabbit IgG, HRP‐linked	Cell Signaling Technology	Goat; unknown	1/2000	AB_2099233
Rabbit IgG		Goat Anti‐Rabbit IgG Alexa Fluor^®^ 594	Abcam	Goat; polyclonal	1/150	AB_2650602
Mouse IgG H&L (DyLight^®^ 488)		Donkey Anti‐Mouse IgG H&L (DyLight^®^ 488) secondary antibody	Abcam	Donkey; polyclonal	1/50	AB_10698084

This table provides details on the antibodies used for immunohistochemistry, immunocytochemistry, and western blotting

### Immunocytochemistry and Immunohistochemistry

Primary stomach culture cells (1 mL of culture media) were plated at a concentration of 1,000,000 cells per mL on sterile glass cover slips in a 12 well cell culture dish. After 24 h in culture, wells were washed 3 × 5 min with TBS containing 4% paraformaldehyde (PFA) for 20 min at room temperature. Cells were washed again 3 × 5 min with TBS. Cells were then treated with 500 *μ*L per well of a permeabilizing/blocking solution (5% normal donkey serum (NDS), 0.1% Triton X‐100 in TBS) for 30 min.

Paraffinized fundus tissue sections of mouse C57 stomach were purchased from Zyagen (San Diego, CA). Tissue sections were deparaffinized as indicated in (Gagnon et al. 2009) excluding the microwave antigen retrieval.

Primary antibodies for ghrelin and CSE were added to the cover slips and tissue sections and incubated overnight in blocking solution at 4°C as indicated in Table 1. Cells and tissue were washed three times in TBS and then incubated in secondary antibodies (Table 1) in blocking buffer for 45 min in the dark. After 3 × 5 min washes in TBS, the cover slip was placed cell‐side down into Fluoroshield Mounting Medium with DAPI (Abcam, Toronto, Canada) and fixed using clear nail polish. The cells were observed in a dark room using an inverted Zeiss Axioplan fluorescent microscope and images were captured using the Zeiss AxioVision software (Zeiss, Oberkochen, Germany).

### In vivo H_2_S experiments

Separate experiments were conducted for the H_2_S inhibitor (PPG) and donor (GYY4137) studies. Following a week‐long acclimation period in the animal care facility, male and female mice were divided into control and treatment groups. All animals were fasted overnight (16 h) to ensure high basal ghrelin levels. For CSE inhibitor experiments, PPG (30 mg/kg body weight) or saline was injected intraperitoneally (IP) 16 h prior to glucose delivery, while the H_2_S donor GYY4137 (30 mg/kg body weight) or saline was IP‐injected 30 min prior to glucose delivery. Animals then received an oral glucose gavage (2 g/kg body weight in water) to elicit a nutrient‐induced drop in circulating ghrelin (Callahan et al. 2004). A small prick was made in the saphenous vein of the animal and blood was collected into EDTA coated capillary tubes for ghrelin measurements at 0, 30, and 60 min timepoints after the oral glucose gavage. After 60 min blood collection, preweighed food was returned and consumption was measured at 1, 2, 3, 4, and 24 h timepoints. Blood samples were stored on ice and centrifuged at 6000*g*, 4°C for 6 min. 20 *μ*L of plasma was used in an enzyme immunoassay kit for acylated ghrelin.

### Ghrelin assays

Ghrelin levels in cell culture and plasma samples were determined using an acylated ghrelin enzyme immunoassay kit (Cayman Chemical) as per manufacturer's guidelines. Dried media and lysate samples were resuspended in immunoassay buffer immediately before assay. Ghrelin levels were determined for entire media and cell lysate from each sample. Results for various treatments were presented as a percent secretion (media/media + cell) relative to untreated control. Acylated ghrelin was analyzed using 20 *μ*L of plasma diluted in assay buffer.

### Data analysis

All data are expressed as mean ± SEM. Studies comparing two groups were analyzed by student's *t*‐test. Studies with multiple doses of the same treatment were analyzed by one‐way ANOVA, followed by a Bonferroni post hoc test. Studies with two independent variables were analyzed by two‐way ANOVA, followed by a Bonferroni post hoc test at individual time points where applicable. *P* < 0.05 was considered significant.

## Results

### The H_2_S donor GYY4137 suppresses ghrelin secretion in vitro

**Figure 1:**
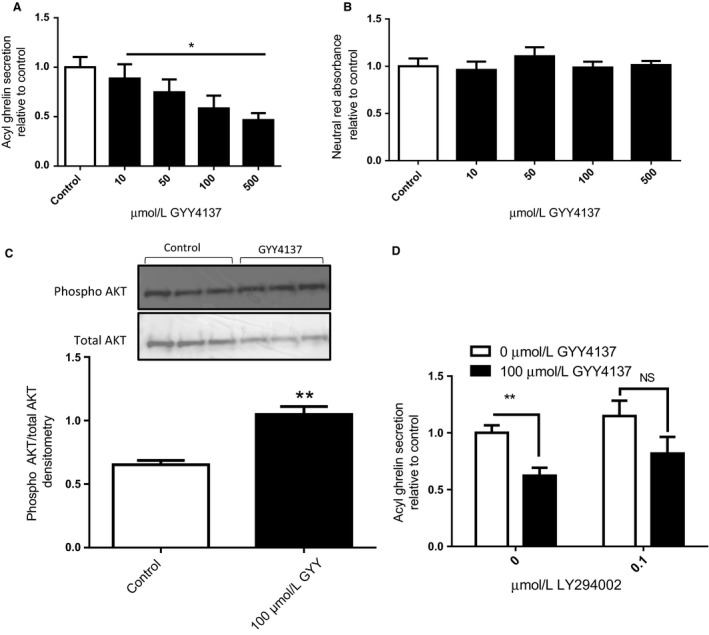
H_2_S suppresses ghrelin secretion in gastric primary culture. Percent acyl ghrelin secretion (A) and neutral red absorbance (B) relative to control was examined after 4‐h treatments with GYY4137. Phosphorylated AKT and total AKT were analyzed using a western blot in cells treated for 15 min with GYY4137 (C). Percent acyl ghrelin secretion relative to control was examined after 4‐h treatments with GYY4137 and/or the PI3K inhibitor LY294002 (D). *n* = 3–6; *P < 0.05 versus control cells, **P < 0.01 versus control cells.

To determine the direct effect of H_2_S on ghrelin secretion, a slow releasing H_2_S donor, GYY4137, or control media were applied to a rat stomach primary culture for 4 h. The mean total of secreted ghrelin from primary culture controls was 146.9 ± 15.58 pg/mL. Overall, GYY4137 decreased ghrelin secretion (*P* < 0.05 overall one‐way ANOVA), with the highest dose (500 *μ*mol/L) causing the largest decrease (0.47 ± 0.07 fold of control Fig. 1A). Cell viability examined by the neutral red assay was not affected by GYY4137 treatments (Fig. 1B).

As previous studies have demonstrated a role for the AKT pathway in ghrelin suppression (Gagnon and Anini 2012), we next investigated AKT phosphorylation in rat stomach primary cultures treated with GYY4137. GYY4137 caused a significant increase in the levels of phosphorylated/total AKT compared to control (*P* < 0.01, Fig. 1C). To determine if the AKT pathway is required for GYY4137‐mediated ghrelin suppression, we coincubated cells with GYY4137 and the PI3K inhibitor LY294002. In these experiments, the effect of GYY4137 was not completely blocked by LY294002 (no statistical interaction); however, the significant suppression of ghrelin secretion normally seen with GYY4137 alone is lost in the presence of 0.1 *μ*mol/L LY294002 (*P* < 0.01 in post hoc analysis, Fig. 1D).

### Endogenous H_2_S regulates ghrelin in vitro

**Figure 2:**
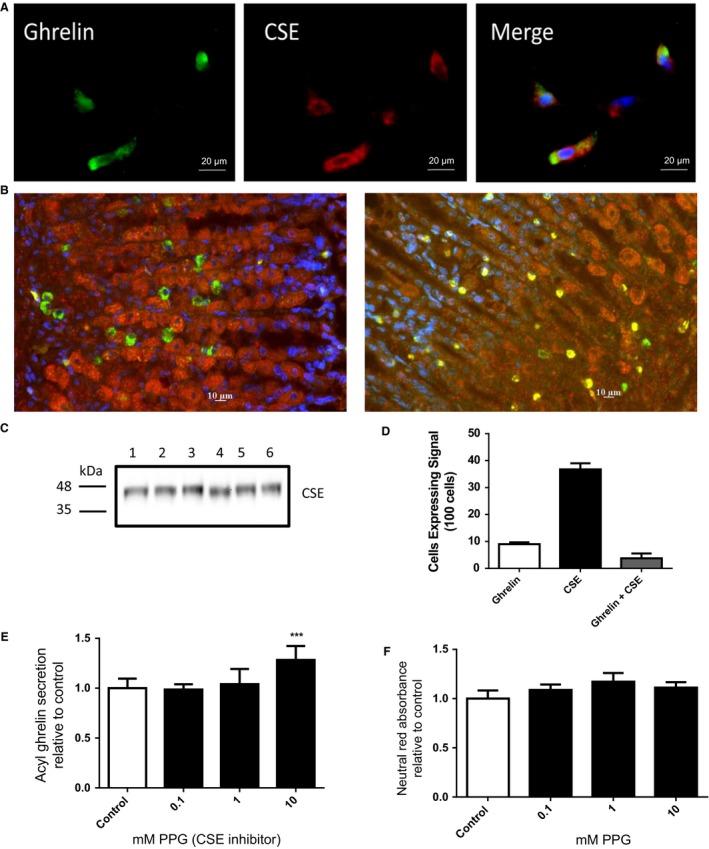
Inhibition of CSE stimulates ghrelin secretion. Primary stomach culture and stomach funds tissue were examined by fluorescent immunocytochemistry/immunohistochemistry for ghrelin (green), CSE (red), and nuclei (blue, in merge) (A and B). CSE MW was confirmed by western blot from primary culture cell lysate *n* = 6 (C). Cells expressing ghrelin, CSE or both were counted in a 100 cell preparation to determine abundance and location within the fundus (D). Acylated ghrelin secretion from primary stomach culture was examined after 4‐h treatments with the CSE inhibitor PPG (E). Cell viability was examined under similar conditions (F). *n* = 6–9; **P < 0.001 versus control cells.

Since exogenous H_2_S caused a suppression in ghrelin secretion, we next examined the role of endogenous H_2_S in ghrelin regulation. To ensure the rat stomach primary culture was a suitable model to study endogenous H_2_S production, we examined the protein expression of the H_2_S‐synthesizing enzyme CSE using fluorescent immunocytochemistry. CSE was expressed throughout the primary culture preparation (Fig. 2A). As such, all ghrelin‐positive cells in the primary culture preparation also coexpressed CSE (Fig. 2A). We then examined mouse stomach fundus using immunohistochemistry to examine abundance and distribution of ghrelin and CSE expressing cells (Fig. 2B). Cellular expression of cells producing ghrelin, CSE or colocalized expression were determined from a count of 100 cells with 9 ± 1 positive for ghrelin, 37 ± 2 positive for CSE and 4 ± 2 colocalized for both (Fig. 2D). To ensure the fluorescent signal for CSE was specific to this enzyme, we completed a western blot of total cell lysate using the anti CSE antibody and detected a signal band at 44 kDa (corresponding to the correct molecular weight of CSE, Fig. 2C).

As we observed a high level of CSE signal in the primary culture preparation, we next determined the effect of inhibiting this enzyme on ghrelin secretion. Cells incubated with the CSE inhibitor PPG had an overall increase in ghrelin secretion (*P* < 0.001 one‐way ANOVA), with the highest dose (10 mmol/L) being 1.28 ± 0.06 fold of control (Fig. 2E) without any effect on cell viability as shown in the neutral red assay (Fig. 2F).

### In vivo effects of H_2_S on postprandial ghrelin levels

**Figure 3:**
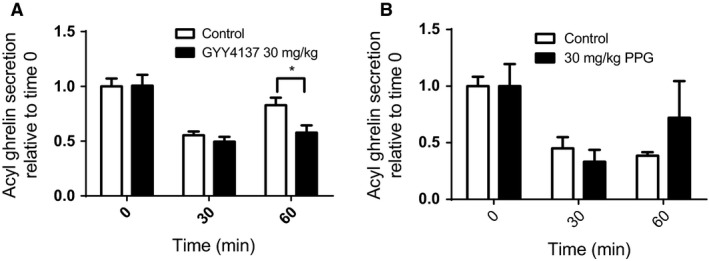
In vivo effects H_2_S on postprandial ghrelin suppression. Plasma acyl ghrelin was measured in mice receiving single injection of the H_2_S donor GYY4137 (A) or the CSE inhibitor PPG (B) at 0, 30 and 60 min after and given an oral glucose gavage. *n* = 14–15 per group; *P < 0.05 versus control animals.

All animal experiments were completed using both male and female mice. Since no statistically significant difference was observed between sex in all parameters investigated, we combined the male and female datasets for data analysis. To determine if the reduction in ghrelin secretion seen in the primary culture with the H_2_S donor GYY4137 would translate to the vivo model, mice were given an IP injection containing either saline or 30 mg/kg GYY4137 prior to the glucose gavage. The mean basal (time 0) plasma ghrelin levels of mice were similar with 77.24 ± 4.35 pg/mL in the control group and 87.88 ± 8.23 pg/mL in the treatment group. While no difference in circulating ghrelin levels were observed at time 0 and 30 min between treatment and controls, GYY4137 significantly prolonged the postprandial ghrelin suppression at 60 min with 0.83 ± 0.07 fold suppression for control and 0.59 ± 0.07 fold suppression for treatment (Fig. 3A, *P* < 0.05). Overall this treatment effect was similar in Male and Female mice (Control 60 min: 0.90 ± 0.12, Female 0.75 ± 0.06, and treatment 60 min: Male 0.55 ± 0.07, Female 0.62 ± 0.13).

To determine if the increase in ghrelin secretion seen in our primary culture with the CSE inhibitor would translate to the in vivo model, mice were given an IP injection containing either saline or 30 mg/kg PPG prior to the nutrient gavage. While a trend of increased ghrelin levels was seen 60 min after glucose in treated animals (Fig. 3B), this was not statistically significant (*P* > 0.05 two‐way ANOVA treatment effect).

### In vivo effects of H_2_S on food consumption

**Figure 4:**
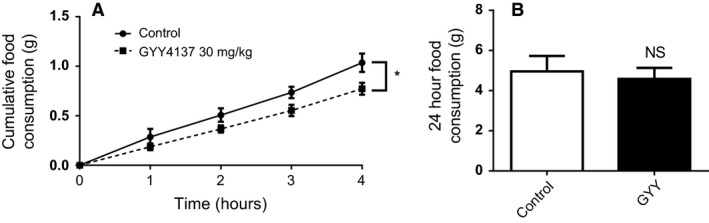
H_2_S reduces short term food consumption in mice. Cumulative food consumption was examined in fasted mice after a single i.p. injection of the H_2_S donor GYY4137 up to 4 (A) and 24 h (B). *n* = 14–15 per group; *P < 0.05 versus control animals.

Since GYY4137 prolonged postprandial ghrelin suppression in mice, we next evaluated its impact on food consumption. GYY4137 caused a significant reduction in cumulative food consumption (*P* < 0.05 for treatment effect) with the 4 h time point having the greatest difference at 1.04 ± 0.09 g in control (Male 1.02 ± 0.08, Female 1.04 ± 0.18) versus 0.77 ± 0.06 g in treatment (Male 0.85 ± 0.09, Female 0.69 ± 0.07) (Fig. 4A, *P* < 0.01 post hoc test). When total food consumed was examined at the 24 h mark, no difference in food consumption was observed (Fig. 4B). Similar to the lack of effect observed with PPG for ghrelin secretion, no effect was observed with PPG for food consumption (data not shown).

## Discussion

In this study, we examined the role of H_2_S in the regulation of ghrelin secretion and appetite. This was investigated through a combination of cell and animal‐based studies using a H_2_S donor, as well as an inhibitor of endogenous H_2_S production.

Using primary stomach culture, we determined that the H_2_S donor GYY4137 causes a suppression in ghrelin secretion and an increase in the phosphorylation of AKT. While other H_2_S donors are available including Na_2_S and NaHS, we selected GYY4137 as it can maintain a steady H_2_S concentrations for the length of both our primary culture (4 h) and in vivo experiments (Li et al. 2008). Our finding of increased pAKT/total AKT signal with reduced ghrelin secretion is in agreement with previous rat primary culture studies delineating the mechanism of insulin‐induced ghrelin suppression (Gagnon and Anini 2012). It should be noted that the reduced pAKT/AKT ratio in this study may have been primarily due to reduced total AKT rather than increased pAKT. A possible mechanism for this reduced total AKT may be through H_2_S induced vascular endothelial growth factor (VEGF) suppression (Merighi et al. 2012), as lowered VEGF is known to increase AKT ubiquitinylation and proteasomal degradation (Riesterer et al. 2004). Further work exploring this potential pathway in gastric cells will resolve this. Nevertheless, other groups have demonstrated the ability of H_2_S to increase phosphorylated AKT levels in cancer cells (Wu et al. 2017) and endothelial cells (Cai et al. 2007), the latter being a required pathway for the proangiogenic effects of H_2_S. When we coincubated the primary culture with the H_2_S donor and the kinase inhibitor LY294002, a partial loss of ghrelin suppression was observed. However, our analysis did not demonstrate significant interaction for LY294002, which suggests that pathways other than PI3K/AKT may be involved in H_2_S‐mediated ghrelin suppression.

Despite efforts, we were unable to quantify endogenous H_2_S production from the primary stomach culture using the methylene blue method (data not shown). This is likely due to the comparatively low levels of expression of H_2_S producing enzymes in the stomach as opposed to the liver (Ishii et al. 2004).

When examined in vivo, GYY4137 caused lower levels of circulating ghrelin 60‐min postprandially. The typical drop and subsequent rise at 60 min has been observed in other ghrelin studies in rodents and humans (Tschöp et al. 2000; Kim et al. 2007). The ability of the H_2_S donor to keep postprandial ghrelin levels lower may lead to reduced feeding. Indeed, this delayed rise in ghrelin coincided with less food consumption over the next 4 h. It should also be mentioned that while we predict that reduced ghrelin levels are responsible for the reduced food consumption in this treatment, additional experiments using ghrelin‐deficient mice would clarify this. This gap in food consumption between treatment and control eventually closed, as there was no difference in total food consumed at the 24 h time point. Future work examining daily delivery of H_2_S donors will clarify whether prolonged food consumption effects are possible.

CSE and cystathionine‐*β*‐synthase (CBS) are the predominant H_2_S producing enzymes in the body, although CBS is mainly expressed in the central nervous system (Abe and Kimura 1996; Robert et al. 2003). As previous work demonstrated that inhibition of CSE reduced nearly all gastric H_2_S production (Fiorucci et al. 2005), we focused our examination of endogenous H_2_S on CSE. We found CSE immunofluorescence throughout the stomach primary culture preparation. Expression was not restricted to ghrelin producing cells, however, all ghrelin producing cells were positive for CSE. This suggests that the stomach possesses the ability to generate H_2_S, and that ghrelin producing cells may be regulated by this gas in both an autocrine and paracrine manner. Furthermore, our detection of CSE enzyme in the stomach is in agreement with previous studies. Early work on CSE demonstrated that while the liver and kidney have the highest levels of CSE expression and activity, the stomach and intestine were the only other organs with detectable levels (Ishii et al. 2004). As the H_2_S donor treatments suppressed ghrelin, we predicted the inhibition of H_2_S production would increase ghrelin. Indeed, when CSE was inhibited with PPG, we observed the expected increase in ghrelin secretion in vitro. Surprisingly, while we did see a trend towards increased 60 min rebound ghrelin levels in the PPG injected mice compared to control, this did not reach significance. It is possible that sampling the animals at 60 min to catch the postprandial ghrelin rebound was insufficient, and that longer time course studies would give a clearer picture. Future work in larger animals (rats) will enable additional blood sampling to resolve this. Alternatively, some recent work has implicated the other predominant H_2_S producing enzyme, CBS, in the stomach (Xiao et al. 2015). An examination of the effects of CBS inhibitors on ghrelin levels would clarify the potential role of this enzyme in ghrelin regulation.

In this study we demonstrated a transient suppression of appetite via a single injection of GYY4137. Future work should investigate if repeated H_2_S delivery can mediate a sustained reduction in appetite. This could be done via chronic delivery of H_2_S donors or diets enriched in precursors for endogenous H_2_S production including cysteine and methionine. Another approach to increasing circulating H_2_S may be through enhancing microbial H_2_S production. Indeed, microbes are responsible for 50–80% of circulating H_2_S (Shen et al. 2013). Our group has demonstrated that sulfur‐containing prebiotics are able to enrich *Desulfovibrio piger* and elevate H_2_S levels in the colon of mice, leading to increased GLP‐1 secretion (Pichette et al. 2017). While in the latter study ghrelin levels were not measured, it is possible that this colonic source of H_2_S could have a significant impact on ghrelin cells.

In conclusion, this study demonstrates for the first time, a role for H_2_S in the regulation of ghrelin and appetite in rodents. Future work will continue to elucidate the mechanisms of H_2_S action on ghrelin producing cells as well as the ability of H_2_S to reduce appetite and stimulate weight loss through the ghrelin system.

## Conflict of Interest

The author reports no conflicts of interest in this work. ES, LW, and JG have nothing to disclose.
